# Quantitative analysis of size and regional distribution of corpora amylacea in the hippocampal formation of obstructive sleep apnoea patients

**DOI:** 10.1038/s41598-021-99795-8

**Published:** 2021-10-22

**Authors:** Cuicui Xu, Jessica E. Owen, Thorarinn Gislason, Bryndis Benediktsdottir, Stephen R. Robinson

**Affiliations:** 1 School of Health and Biomedical Sciences, RMIT University, Bundoora, VIC Australia; 2 Department of Respiratory Medicine and Sleep, Landspitali − The National University Hospital of Iceland, Reykjavik, Iceland; 3 Department of Clinical Biochemistry, Landspitali − The National University Hospital of Iceland, Reykjavik, Iceland; 4 Institute for Breathing and Sleep, Austin Health, Heidelberg, VIC Australia; 5 School of Health and Biomedical Sciences, College of Science, Engineering & Health, RMIT University, PO Box 71, Bundoora, VIC 3083 Australia

**Keywords:** Molecular biology, Neuroscience, Diseases, Pathogenesis, Risk factors, Signs and symptoms

## Abstract

Corpora amylacea (CoA) are spherical aggregates of glucose polymers and proteins within the periventricular, perivascular and subpial regions of the cerebral cortex and the hippocampal cornu ammonis (CA) subfields. The present study quantified the distribution of CoA in autopsied hippocampi of patients with obstructive sleep apnoea (OSA) using ethanolamine-induced fluorescence. CoA were observed in 29 of 30 patients (96.7%). They were most abundant in periventricular regions (wall of lateral ventricle, alveus, fimbria and CA4), rarely found in the CA3 and CA1, and undetectable in the CA2 or subiculum. A spatiotemporal sequence of CoA deposition was postulated, beginning in the fimbria and progressively spreading around the subpial layer until they extended medially to the wall of the lateral ventricle and laterally to the collateral sulcus. This ranked CoA sequence was positively correlated with CoA packing density (count and area fraction) and negatively correlated with CoA minimum diameters (*p* < 0.05). Although this sequence was not correlated with age or body mass index (BMI), age was positively correlated with the mean and maximum diameters of CoA. These findings support the view that the spatiotemporal sequence of CoA deposition is independent of age, and that CoA become larger due to the accretion of new material over time.

## Introduction

Corpora amylacea (CoA) are polyglucosan bodies with a round or oval profile, that have been reported in various organs and species, particularly in the human brain^1,2^. Since 1837 when CoA were first described by Purkinje, various hypotheses have been proposed regarding the origin of CoA^3–5^. In the brain, CoA have been associated with various conditions, such as normal ageing, hippocampal sclerosis, temporal lobe epilepsy, multiple sclerosis, Alzheimer’s disease (AD) and Parkinson’s disease^1,6–8^. CoA are rarely seen in younger human brains, while increased numbers can be detected after the fourth decade of life^1^; and they are reported to become larger, although this has not been confirmed with quantitative measurements^1^. CoA are observed primarily in periventricular, perivascular, subpial and subependymal regions^2,9–11^. There are abundant blood vessels and astrocytic processes in the subpial and subependymal regions, and CoA distribution has been closely associated with blood vessels and cerebrospinal fluid (CSF)^10^, and CSF transudate has been postulated to contribute to CoA growth^10^.

The numbers of CoA in the human brain appear to increase with age. For instance, a study of ventroposterolateral nucleus of the thalamus observed no CoA in patients younger than 50, and thereafter the numbers and size of CoA appeared to increase with age, although this relationship was not analysed statistically^12^. A second study counted CoA in 51 non-glaucomatous human eyes (aged 2.5–78 years), and reported that CoA count showed a positive linear correlation with age (*r* = 0.424, *p* < 0.01)^13^. Finally a trend of increased CoA count with advancing age was observed in the spinal cord, but some aged subjects had small numbers of CoA and the correlation between number of CoA and age did not reach statistical significance^14^.

The diameters of CoA in the human retina and optic nerve range from 3.6 to 11.0 µm^13^, while in the anterior horn grey matter of spinal cord, most range between 4 and 12 µm, with few exceeding 20 µm^14^. Recent studies have distinguished immature CoA from mature CoA^3,15^. Immature CoA are intracellular inclusions that are enclosed by the cytoplasmic membrane of a cell (primarily in perivascular glial cells), with diameters typically less than 10 µm^15^, but they do not contain a membrane of their own^3^. By comparison, mature CoA are extracellular, are more abundant, can measure up to 30 µm in diameter, and are surrounded by myelin sheaths and cells^15^. These recent reports focused on the composition of CoA in 3–6 aged patients^3,15^, and the sample sizes were too small to permit an assessment of whether the diameters of CoA increase as a function of age.

In the hippocampus, CoA have been observed beneath the pial border, adjacent to the lateral ventricle^1,16^. Cherian and colleagues (2003)^17^ developed a 4-stage grading system to compare CoA density in hippocampal subregions from patients with mesial temporal lobe epilepsy. They reported that CoA were more abundant in CA1 and CA3 than in the dentate gyrus or CA2, and speculated that the density of CoA might correlate with neuronal loss.

CoA have been widely reported to be located in proximity to blood vessels^2,7,10,15,16,18^. In the only quantitative study regarding the association between CoA and blood vessels, CoA were reported to congregate in the perivascular spaces and adjacent neuropil, with 37% of CoA being located within 5 µm of a blood vessel and 77% within 10 µm^15^. This pattern of distribution is different from that expected due to chance and may indicate that CoA have a functional relationship with blood vessels.

A feature lacking from all studies of CoA is that none have systematically measured the packing density of CoA or compared CoA in different subregions of the brain in a quantitative way. Almost two centuries have elapsed since they were discovered, yet we still lack accurate estimates of the density and size of CoA at different ages, and we do not know whether their spatial distribution is random or conforms to a pattern. Furthermore, systematic observations are not available concerning the spatial relationships between CoA and the CSF or blood vessels. If such information were available, it might help to inform speculation on the origins of these enigmatic structures.

The present study was conceived through the serendipitous observation of fluorescent CoA while conducting a larger study of neuropathological changes in the hippocampi of people with OSA^19,20^. It was observed that CoA appeared in similar locations in many of the samples and this provided the opportunity to systematically map their distribution. The present study seeks to fill major gaps in our knowledge of CoA by mapping the distribution and size of CoA in hippocampal subregions of OSA patients, and relating these parameters to patient age.

## Methods

### Ethics approvals

This project was approved by the Department of Pathology Biobank, University Hospital, Reykjavic (permit number L07/13), and the National Bioethics Committee, Iceland (reference number 09-087-CM) for the autopsy brain tissue samples (formalin-fixed and paraffin-embedded) and corresponding medical records to be examined at RMIT University, Melbourne, Australia. The research investigations conducted on these samples were approved by the RMIT Human Research Ethics Committee (reference number ASEHAPP 71-16).

### Study sample

The hippocampal series consisted of autopsy tissue from 30 OSA patients (16 males and 14 females) from Iceland who had died between 1987 and 2014, between the ages of 41.7–89 years, with the average age at death being 67.5 years. As this study is a part of a larger project, additional details of this sample can be found in previous publications^19,20^. Briefly, formalin-fixed, paraffin embedded tissue was shipped to RMIT University, brain blocks that included the wall of lateral ventricle, fimbria, parahippocampal gyrus and collateral sulcus were selected and sectioned coronally at 20 µm.

### CoA identification

CoA were initially identified in sections stained for anti-tau^20^, cresyl violet^19^ and anti-GFAP. It has been previously established that CoA can be identified with these staining methods^21,22^. It was then discovered that the same structures appear as bright green-yellow profiles when fully processed control slides were illuminated with blue light and observed under the fluorescent illumination (FITC channel, emission wavelength 518 nm, 500 ms exposure time). A comparison image of the same hippocampus stained with cresyl violet, anti-tau, anti-GFAP and in a fully processed control slide viewed with fluorescence illumination has been included in the supplementary information (Supplementary Fig. S2).

When we first considered the identity of the profiles, we ruled out Gomori-positive astrocytic inclusions because Gomori inclusions fluoresce red but not yellow, they are angular or irregular in profile (not spherical), and they are small (0.5–10 μm in diameter, with most being less than 2 μm)^23^. Similarly, we excluded lipofuscin, since this is visible as a brown deposit under transmitted illumination, is granular and generally outlines the neuronal soma in which it is located^24,25^. Indeed, we observed both Gomori-positive astrocytic inclusions and lipofuscin inclusions in neurons in our tissue, and took care not to include these in our analysis.

The sections used for CoA visualization were control slides from immunohistochemistry performed as part of a previous study^19,20^. These control slides had been processed in an identical manner to the immunocytochemistry slides, except for the primary antibody, which had been omitted. The processing protocol is detailed in the following section.

It should be noted that CoA do not autofluoresce, and we were unable to visualise CoA in unprocessed control sections. Through a process of elimination, we established that the induction of fluorescence in CoA depends on the inclusion of ethanolamine in the blocking step. Ethanolamine, in the presence of trace amounts of citrate or borate, causes carbohydrates and monosaccharides to fluoresce^26,27^, and we speculate that the citrate present in BSA^28^ interacts with ethanolamine to cause the polyglucosans in CoA to fluoresce.

### Immunohistochemistry

Sections were deparaffinised with histolene (2 × 5 min) and graded ethanol solutions (100% 3 min, 95% 3 min, 70% 3 min, distilled water rinse 1 min). Sections were then incubated with blocking solution (0.1 M phosphate buffered saline (PBS), 1% BSA, 4% serum, 1% Triton X-100 and 1% ethanolamine) for 3 h at room temperature. Note serum from the host animal of the secondary antibody were used to reduce non-specific labelling. Tissue sections were incubated with solutions (0.1 M PBS, 1% BSA, 4% serum and 1% Triton X-100) containing primary antibodies in a humidified chamber for 18 h at room temperature overnight. On the next day, sections were collected out of the container and then incubated for 3 h with solutions (0.1 M PBS, 1% BSA and 4% serum) of secondary antibodies diluted at 1 in 300. Between each of the above steps, sections were washed for 3 × 10 min in 0.1 M PBS. After tertiary incubation with a solution (0.1 M PBS and 1% BSA) of streptavidin-biotinylated horseradish peroxidase (SB-HRP) diluted at 1 in 300 for 3 h, sections were washed for 2 × 5 min in 0.1 M PBS then for 2 × 10 min in 0.175 M sodium acetate buffer (NaOAc). Sections were then incubated for 10 min in a 0.05% solution of the chromogen DAB diluted in NaOAc buffer, then in 0.05% DAB in NaOAc buffer with 0.004% H_2_O_2_ for another 15 min to visualise the chain of antibodies bound to the tissue. Immunolabelled sections were then washed in NaOAc buffer for 2 × 5 min then 0.1 M PBS for 2 × 10 min and left overnight in 0.1 M PBS at 4 °C. On the following day, sections were dehydrated in graded ethanol (70% 10 min, 95% 10 min, 100% 2 × 5 min) and histolene (2 × 10 min) before being coverslipped with Depex. The immunohistochemistry diluents are summarised in supplementary Table S1.

Immunohistochemistry staining with a cocktail of two primary antibodies, with DAB as the chromogen for both, was used to visualize entire blood vessel segments rather than one part of the blood vessel alone. The protocol was altered slightly from that described previously^20^. Briefly, after deparaffinization and blocking, sections were co-incubated with both antibodies of anti-Collagen IV (Rabbit serum, Abcam, ab6586, 1 in 20,000) and anti-GluT-1 (Rabbit serum, Abcam, ab15309, 1 in 2,000) followed by incubation with appropriate secondary antibody (Goat anti-Rabbit, Vector, BA-1000, 1 in 300) followed by streptavidin-biotinylated horseradish peroxidase (SB-HRP, GE Healthcare, RPN1051, 1 in 300) and a solution of diaminobenzidine and hydrogen peroxide was used as the chromogen.

### Image processing

Sections were viewed with an Olympus fluorescence microscope (VS-BX) and camera (Hamamatsu ORCA-Flash 4.0), using a ‘Fluorescein’ filter set (emission wavelength 518 nm, filter wheel (reflected) 485, and filter wheel (reflected) 525). Scanning images were acquired with an Olympus Virtual Microscopy Slide Scanning System (VS120) and Olympus VS-ASW-S5 software, FITC (Fluorescein isothiocyanate) channel at 500 ms exposure time, final magnification of 20 × (objective lens 20 × , camera adapter mag 1 ×), automatic Z-stacking (20 µm range, 4 µm step size, therefore 5 planes in total). Images to be used for analysis were captured from the full view images at a resolution of 1600 × 1200 pixels (equivalent to 0.2 mm^2^) at pre-determined locations. The coloured images were converted into grayscale, rendered as black/white images in Adobe Photoshop CS6 Extended, and then analysed with Olympus CellSens software. The minimum detection size was equivalent to 3 µm. Objects that intersected the left hand or bottom borders of the image were excluded from analysis. All brain sections were processed in an identical manner.

Seven hippocampal neuropil regions that surround the lateral ventricle were investigated in the present study (Supplementary Fig. S1A), including the wall of lateral ventricle (LV), fimbria, cornu ammonis 4 (CA4), cornu ammonis 3 (CA3), cornu ammonis 1 (CA2), cornu ammonis 1 (CA1), and subiculum. In addition, the subpial regions in the following locations were investigated (Supplementary Fig. S1B): alveus pial surface (APS), fimbria pial surface (FPS), prosubiculum pial surface (PPS), subiculum pial surface (SPS), medial entorhinal cortex pial surface (MPS), lateral entorhinal cortex pial surface (LPS) and collateral sulcus pial surface (CPS). These regions were chosen because they are spaced across the hippocampal formation, and can be reliably identified due to their proximity to landmarks.

### CoA detection and quantification

**Figure 1:**
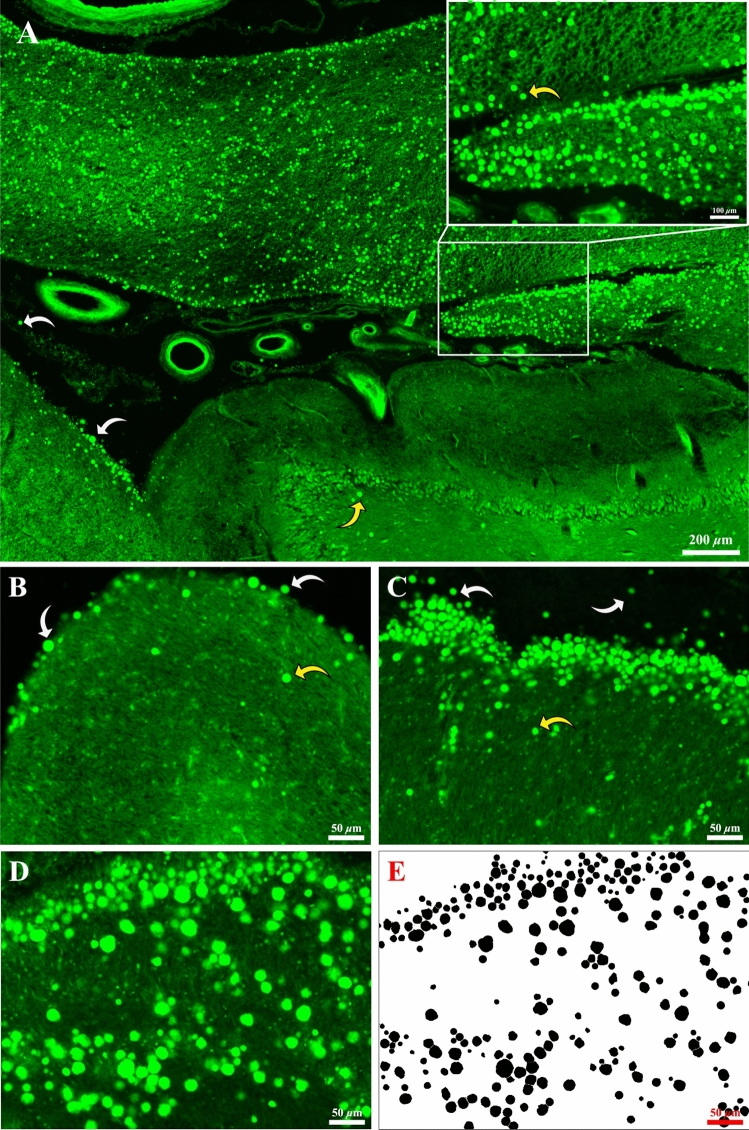
(**A**) Representative low-mag CoA distribution micrograph under fluorescent mode (FITC) in the fimbria region. Inset was captured with the ‘Crop’ tool in Olympus CellSens software at a resolution of 8000 × 6000 pixels. Individual CoA are indicated by arrows. Higher magnification micrographs (1600 × 1200 pixels) (**B**,**C**) show aggregations of CoA beneath the pial surface (yellow arrows), while some CoA are observed within the cavity of the ventricle or straddling the pial surface (white arrows). Representative demonstration of original micrograph (**D**) and the corresponding rendered image (**E**) in the fimbria region generated with Photoshop software for data collection and analysis (1600 × 1200 pixels). Note the demonstrated micrograph was the 3rd plane of a 5-step Z-stack (Z range 20 µm, step size 4 µm), the upper and lower step images were referred to in order to generate the processed image. Therefore, in some cases minor mismatching occurs between the processed image and the single slice immunofluorescent micrograph.

When viewed under fluorescent illumination (Fig. 1A), CoA appeared as bright green circles against the pale background autofluorescence of the tissue, and were concentrated in the vicinity of the fimbria. CoA were sometimes observed on the pial surface (Fig. 1B,C), where they appear to be located within the ventricular cavity (white arrows), rather than in the neuropil (yellow arrows). For the purpose of data collection and analysis, the original micrographs were processed with Photoshop software. In rendered images CoA appear as filled black circles on a white background (Fig. 1D,E), which were quantified with Olympus CellSens software for five parameters: (1) CoA count, the total number of CoA per field of view; (2) CoA area fraction (%), the proportion of a field of view occupied by CoA; (3) CoA mean diameter (µm), the average diameter of all CoA in a field of view; (4) CoA minimum diameter (µm), the mean diameter of the smallest CoA in a field of view; (5) CoA maximum diameter (µm), the mean diameter of the largest CoA in a field of view. In some cases, no CoA were detected in a field of view, in such cases a ‘0’ value was recorded for the CoA count and area, and this value was used in the analysis. However, for CoA diameter, values of 0 µm were not included in the analysis.

### Definition and classification of CoA stages

In previous research, the areal density of CoA within specific brain regions has been graded according to the semiquantitative criteria of Cherian et al.^17^ that are based on high-magnification images of a small area. In the present study we observed that CoA are not randomly distributed, and hence the use of high magnification images provides variable and unrepresentative estimates of CoA density. In order to obtain more accurate estimates, the present study examined CoA density in lower magnification images (20 ×) that encompassed a much larger area (0.2 mm^2^) of tissue than typically assessed. Therefore, new criteria needed to be developed to classify the stages of CoA based on the density of CoA in specific regions, as well as considering the spatial distribution of CoA across an entire section.

### Full view diagrams of CoA distribution in the hippocampus

In order to stage individual brains, it was necessary to first ascertain the distribution and relative frequency of CoA across the hippocampus. Full view scanned images of each brain section were processed with Photoshop software to generate outline diagrams of the section (please refer to supplemental data).

### Quantification of CoA in high-density and low-density zones

On inspection of the sections it was observed that the density of CoA peaks just beneath the pial border; there is a fairly distinct boundary between the high-density subpial zone and the deeper layers of tissue that have a low density of CoA. We have termed these two regions ‘high-density zone’ (HD zone) and ‘low-density zone’ (LD zone) respectively. The HD zone was enclosed with a ‘polygon’ and another line was drawn along the centre of the enclosed area, parallel to the pial surface, in Olympus CellSens software; the HD zone depth was estimated as the enclosed area divided by the length of the centre line.

### Statistical analysis

Statistical analysis was performed with IBM Statistical Package for the Social Sciences (SPSS version 21). Unpaired two-tailed student’s *t*-tests were used to compare the sets of data between two groups. One-way ANOVAs were performed for comparison between more than two groups, while Levene's test was included as a measure of whether the variances of the compared groups were equal; the LSD post hoc test was used with equal variances, while the Games-Howell post hoc test was used with unequal variances. Spearman's rho correlations were performed for the covariate of two continuous-level variables. False Discovery Rate (FDR) corrections were performed to eliminate the risk of Type 1 error (*i.e.* retention of false positives) due to multiple comparisons. * *p* < 0.05 was considered to be significant with equal variances, alternatively, # *p* < 0.05 indicates significant difference with unequal variances. GraphPad Prism 7 was used to generate graphs, data and figures were present as Mean ± SEM.

## Results

### CoA detection

Seven hippocampal neuropil regions and seven pial surface regions were investigated, CoA were observed in 29 fimbria regions out of 30 (96.7%), and in 23 of 24 brain samples (95.8%) that included the wall of the lateral ventricle (Supplementary Table S2). One case contained no CoA throughout the hippocampus. All of the CoA found in the CA4 region (19 out of 30, 63.3%) were located close to the inferior horn of the lateral ventricle, rather than in the deeper grey matter. Sparse CoA were occasionally observed in the CA3 region (3 out of 30, 10.0%) and CA1 region (4 out of 30, 13.3%), and no CoA were observed in the grey matter of the CA2 or subiculum. CoA were present in all of the seven pial surface regions to varying extents (58.3–96.7%).

### CoA distribution: stage and sequence

When diagrams of CoA distribution were compared, a consistent pattern became evident: in sections with low burdens of CoA, they were concentrated in the vicinity of the fimbria; as the burden increased, CoA extended further away from the fimbria, within the pial border, to the alveus and wall of lateral ventricle on one side, and towards the subiculum, entorhinal cortex and beyond the collateral sulcus on the other side. CoA were only observed in the deep white and grey matter in sections with the heaviest burdens. Based on these diagrams, three investigators (C.X., J.E.O. and S.R.R.) independently arranged the sections in a series, from lowest CoA burden to highest. There was strong concordance between investigators, and the minor differences in the order of the rankings were resolved by discussion. The agreed CoA sequence (1–30) was then divided into five stages, based on the spatial extent of spread of CoA and the severity of the CoA burden (see supplementary data).

Stage 0 corresponded to no CoA being present. Stage 1 was defined as a low density of CoA (0–10 CoA per field of view) in the fimbria, without progression of CoA across the pial surface. Stage 2 was defined as medium density (10–50) CoA in the fimbria and along the pial surface of the prosubiculum and subiculum. Stage 3 was defined as medium or high density (50–500) CoA in the fimbria, and extending continuously across the pial surface of the prosubiculum and subiculum, as well as into the deep white matter of the wall of lateral ventricle. Stage 4 was defined as medium or high density (50–500 CoA); this stage resembled Stage 3, except that CoA now extended into the deep white matter of the parahippocampal gyrus and fusiform gyrus (see supplementary data).

Spearman's Rho correlations between ranked CoA sequence (1–30) and measured CoA parameters (count, area fraction, mean, min. and max. diameters) in seven pial surface regions were conducted to investigate the reliability of the staging criteria. Significant positive linear regressions were found between ranked sequence and CoA density (count and area fraction) in the alveus pial surface, fimbria pial surface, prosubiculum pial surface, subiculum pial surface, medial entorhinal cortex pial surface and lateral entorhinal cortex pial surface (Table 1) after FDR corrections. These correlations support the notion that the subjective criteria applied in the present study are in line with the quantifiable parameters, and the ranked CoA sequence is reflective of increasing CoA burden.

**Table 1:** Spearman's Rho correlation of ranked CoA sequence with tested CoA parameters.

	APS	FPS	PPS	SPS	MPS	LPS	CPS
**CoA count**							
*r*	**0.529**	**0.601**	**0.624**	**0.655**	**0.566**	**0.626**	0.131
*p*	**0.004**	**0.002**	**0.002**	**0.001**	**0.005**	**0.002**	0.542
*n*	**30**	**30**	**30**	**24**	**24**	**24**	24
**Area fraction**							
*r*	**0.555**	**0.620**	**0.668**	**0.763**	**0.657**	**0.600**	0.182
*p*	**0.002**	**0.001**	** < 0.001**	** < 0.001**	**0.001**	**0.002**	0.395
*n*	**30**	**30**	**30**	**24**	**24**	**24**	24
**Mean diameter**							
*r*	− 0.097	− 0.124	0.116	0.061	0.142	− 0.596	0.304
*p*	0.781	0.912	0.770	0.781	1	0.172	0.553
*n*	22	29	29	23	23	14	23
**Minimum diameter**							
*r*	− 0.196	− 0.158	− 0.278	− 0.407	− 0.397	− **0.714**	0.077
*p*	0.535	0.481	0.253	0.188	0.141	**0.029**	0.727
*n*	22	29	29	23	23	**14**	23
**Maximum diameter**							
*r*	0.211	0.271	0.358	0.271	0.466	0.319	0.235
*p*	0.347	0.360	0.199	0.370	0.174	0.373	0.327
*n*	22	29	29	23	23	14	23

Significant differences are indicated in bold.

The listed *p* values are after adjustment for Type 1 error with FDR.

*APS* alveus pial surface, *FPS* fimbria pial surface, *PPS* prosubiculum pial surface, *SPS* subiculum pial surface, *MPS* medial entorhinal cortex pial surface, *LPS* lateral entorhinal cortex pial surface, *CPS* collateral sulcus pial surface.

Significant negative correlations were found between ranked CoA sequence and CoA minimum diameter (*r* = − 0.714, *p* = 0.029) in the lateral entorhinal cortex pial surface region after FDR corrections. Therefore, ranked CoA sequence was inversely correlated with CoA minimum diameters.

### Proposed spatio-temporal pattern of CoA deposition

**Figure 2:**
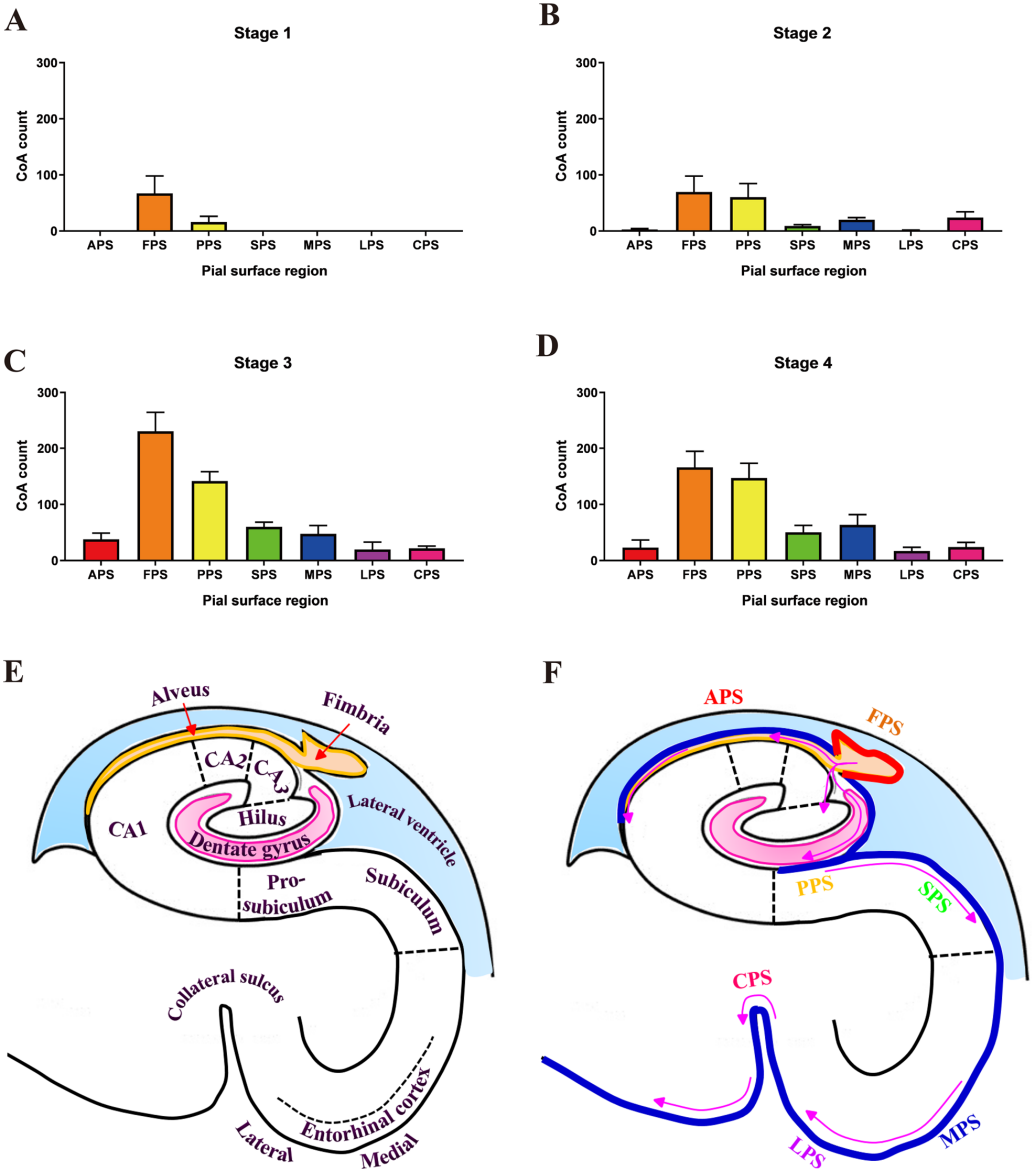
CoA count in seven pial surface regions at stage 1 (**A**), stage 2 (**B**), stage 3 (**C**) and stage 4 (**D**). Mean ± SEM. E, Schematic diagram of the hippocampus, modified from Yang Y, et al. (2008)^40^. F, Proposed progression of CoA along the pial surface; red curve indicates highest CoA density in the fimbria pial surface region; blue curve indicates the decreased CoA density along the pial surface when spreading bilaterally from the fimbria, as pointed by the purple curved arrows. *APS* alveus pial surface, *FPS* fimbria pial surface, *PPS* prosubiculum pial surface, *SPS* subiculum pial surface, *MPS* medial entorhinal cortex pial surface, *LPS* lateral entorhinal cortex pial surface, *CPS* collateral sulcus pial surface.

The progression of CoA along the pial border was investigated in the seven pial surface regions. In stage 1, CoA were restricted to the fimbria (Fig. 2A). By stage 2, CoA were concentrated in the fimbria and prosubiculum (Fig. 2B). In stages 3 and 4 the numbers increased further in the fimbria and prosubiculum, and CoA became evident in the other five pial areas, with a trend to decreasing density with increasing distance away from the fimbria toward the collateral sulcus (Fig. 2C,D). The patterns of CoA distribution in the pial surface were similar in stages 3 and 4, as the main criterion distinguishing these two stages was the presence of CoA in deep white matter.

At every stage, the pial surface of the fimbria contained the most CoA, followed by the pial surface of the prosubiculum in second place. The CoA progression along the pial border started from the fimbria and spread to the alveus, and the corner of lateral ventricle on one side; and towards the prosubiculum, subiculum, entorhinal cortex and collateral sulcus on the other side (Fig. 2E,F) with gradually reduced CoA packing density and size. The mean maximum diameters of CoA display a decreasing trend when progressing from the fimbria pial surface towards the collateral sulcus pial surface, as shown in Table 2.

**Table 2:**
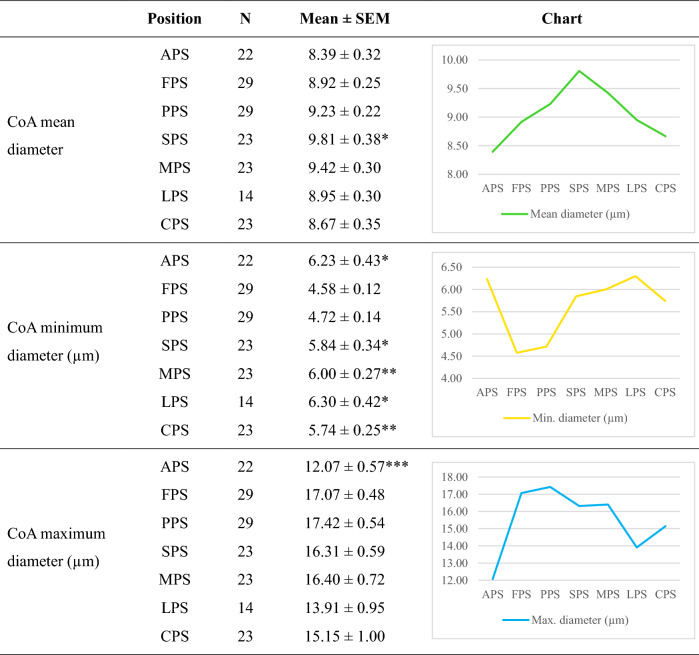
CoA sizes in seven pial surface regions.

CoA at fimbria pial surface (FPS) region display the smallest minimum diameter among all seven pial surface regions, and there is a trend towards a larger size when progressing laterally from the FPS to the CPS; while the maximum diameter show a decreasing trend. One-way ANOVAs with post hoc test for multiple comparisons were performed: FPS compared to APS, PPS, SPS, MPS, LPS and CPS. LSD post hoc test was used with equal variances, while Games-Howell were used with unequal variances. The listed *p* values are after adjustment for Type 1 error with FDR.

*APS* alveus pial surface, *FPS* fimbria pial surface, *PPS* prosubiculum pial surface, *SPS* subiculum pial surface, *MPS* medial entorhinal cortex pial surface, *LPS* lateral entorhinal cortex pial surface, *CPS* collateral sulcus pial surface.

*p < 0.05, **p < 0.01, ***p < 0.001.

### Stratification of CoA within the pial surface region

**Figure 3:**
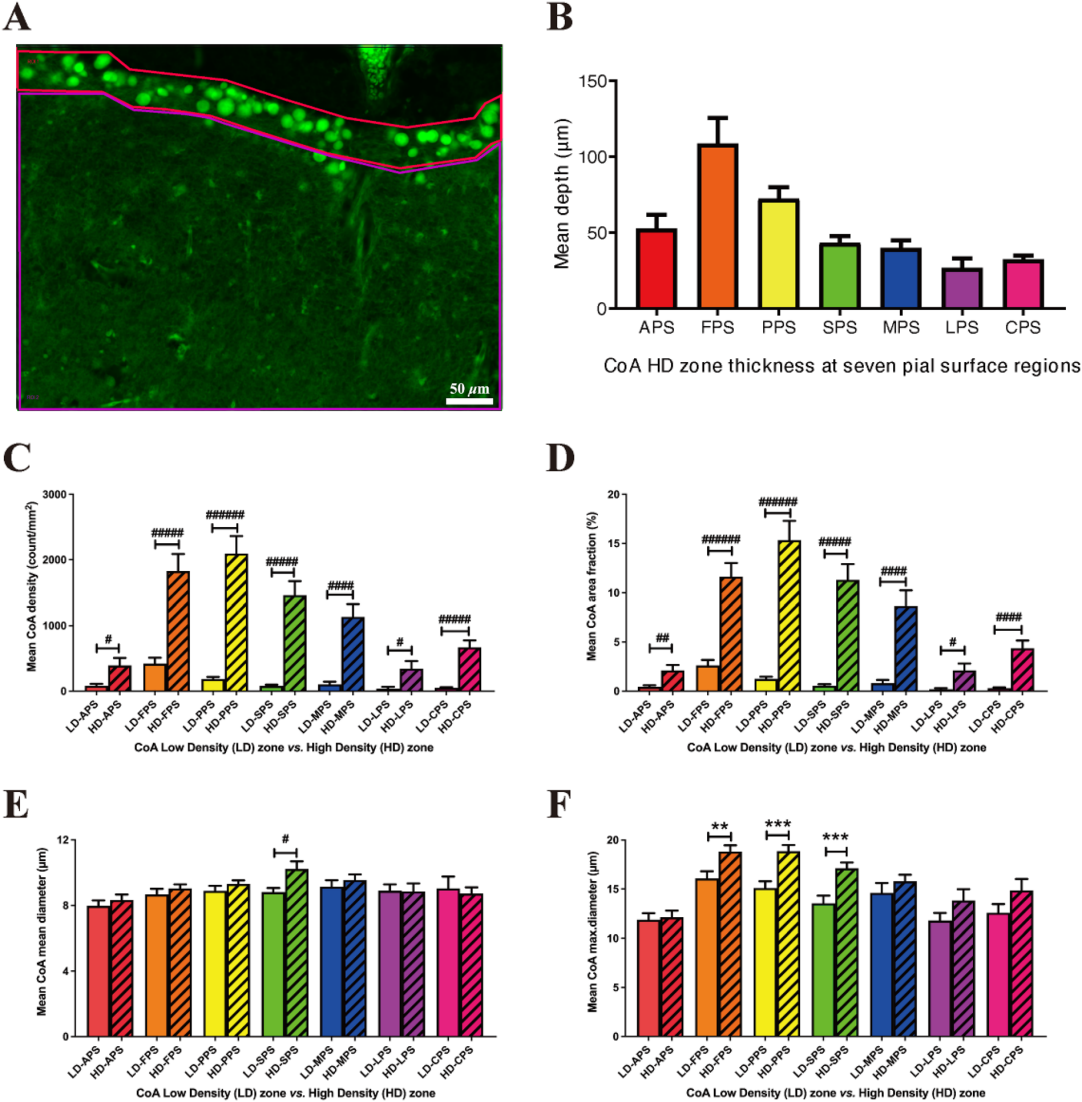
(**A**) Representative CoA high density (HD) zone (top red zone) *vs.* low density (LD) zone (bottom purple zone) at medial entorhinal cortex. (**B**) HD zone thickness (µm) at seven pial surface regions. The difference of CoA burden in the LD zones (blank bars) from those in the HD zones (striped bars), comparing CoA packing density (**C**), area fraction (%) (**D**), mean diameter (**E**) and maximum diameter (**F**). Unpaired 2-tailed *t*-tests. Mean ± SEM. *p < 0.05, **p < 0.01, ***p < 0.001.

Examination of micrographs of the pial region revealed that CoA were concentrated in a well-defined zone just beneath the pial border, which we have termed the ‘high density zone’ (HD zone, top red enclosed zone in Fig. 3A). The mean thickness of the seven HD zones was 52.4 µm at the APS (alveus), 108.4 µm at the FPS (fimbria), 72.2 µm at the PPS (prosubiculum), 43.0 µm at the SPS (subiculum), 39.8 µm at the MPS (medial entorhinal cortex), 26.6 µm at the LPS (lateral entorhinal cortex) and 32.2 µm at the CPS (collateral sulcus) (Fig. 3B). To simplify, for the purpose of quantitative analysis, the HD zone in the present study was defined as extending from the pia to a depth of 100 µm at the fimbria, 70 µm at the prosubiculum and 40 µm below the pial surface at the alveus, subiculum, medial and lateral entorhinal cortex and collateral sulcus.

The deeper region with less CoA was referred to as the ‘low density zone’ (LD zone) and for the purposes of analysis consisted of the remainder of the area in each micrograph (bottom purple enclosed zone in Fig. 3A). Comparisons between CoA packing density (count/mm^2^; calculated as the total CoA count in the region of interest) in the LD zone and HD zone are shown in Fig. 3C. CoA packing density in the HD zones was significantly higher (4.4–18.3-fold) than in the LD zones in all seven regions investigated; moreover, a similar pattern applied to the CoA area fraction (%) comparison, with the HD zones containing 4.4–20.3-fold more area of CoA than the corresponding LD zones (Fig. 3D). The mean diameters (µm) of CoA in the seven HD zones were similar to those in the corresponding LD zones (Fig. 3E), except in the subiculum where CoA had a larger mean diameter (1.2-fold) in the HD zone than that in the LD zone. The CoA maximum diameters in the HD zones were significantly greater (1.2–1.3-fold) than those in the LD zones in the fimbria, prosubiculum and subiculum (Fig. 3F).

### CoA distribution in relation to the microvasculature

**Figure 4:**
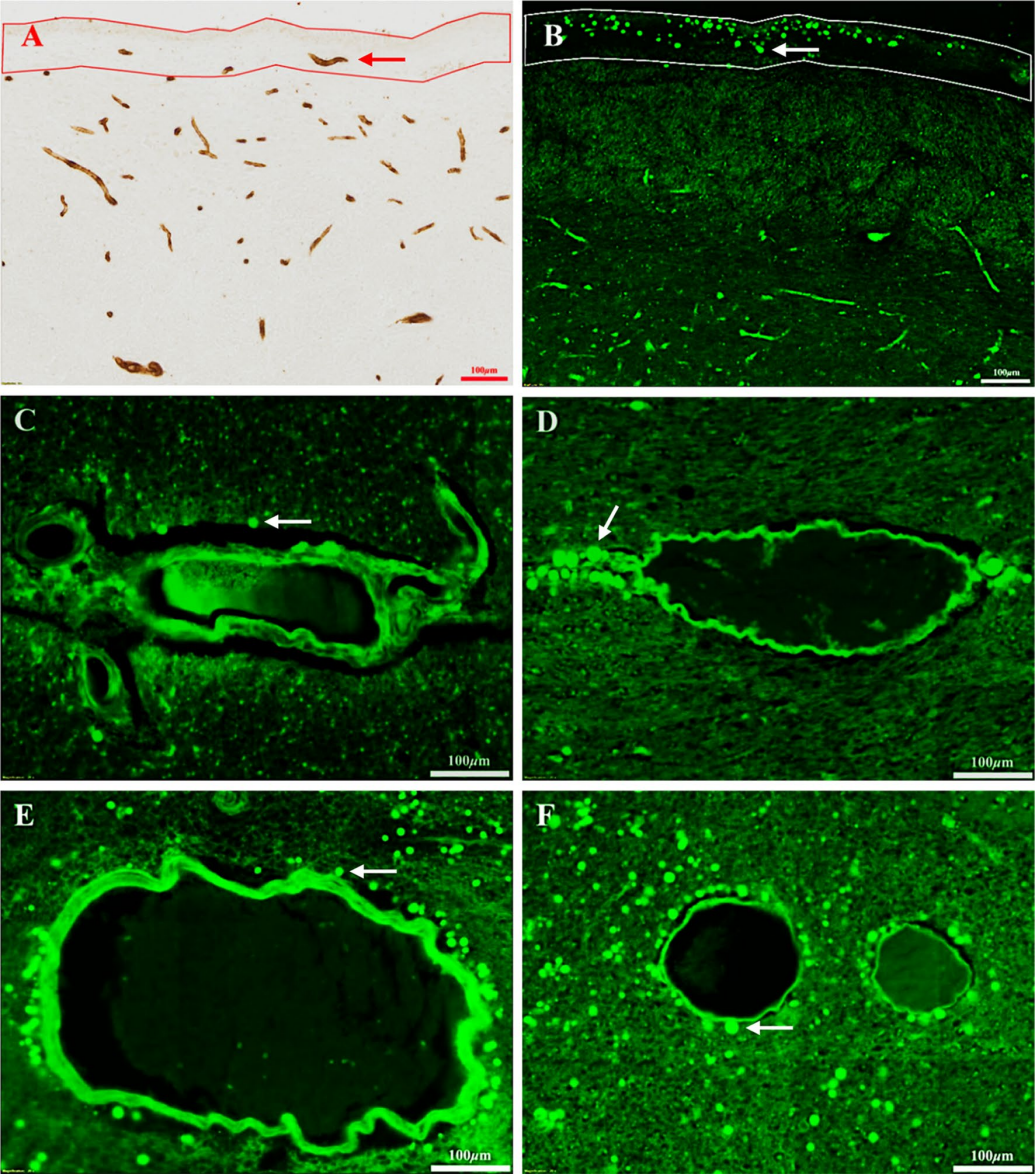
Adjacent brain sections showing the inverse relationship between microvasculature (**A**; immunolabeled with Collagen IV and GluT-1) and CoA (**B**) in the alveus pial surface region. There are very few microvessels within 100 µm of the pial border (avascular zone, red enclosed zone in panel **A**), whereas CoA reach their peak abundance in this zone (white enclosed zone in panel **B**). An example of an individual microvessel is indicated by the red arrow in A, and an individual CoA is indicated by the white arrow in (**B**). The deposition of CoA in relation to arterioles (**C**,**E**) and venules (**D**,**F**) in the deeper neuropil (**C**,**D**) and subpial regions (**E**,**F**). Examples of individual CoA are indicated by white arrows.

The distribution of CoA was compared to the distribution of microvessels, as shown in Fig. 4. Although no quantitative comparison was made, it was evident that very few microvessels are present near the pial surface, and there is a well-defined avascular zone, 100 µm wide, as indicated by the red enclosed zone in Fig. 4A. Interestingly, CoA tend to aggregate in this avascular zone (white enclosed zone in Fig. 4B), suggesting that there is an inverse relationship between the packing densities of CoA and microvessels.

CoA were observed near arterioles and venules in 17 (56.7%) of the 30 OSA hippocampal tissues examined in the present study. However, it needs to be noted that in these 17 specimens, the vast majority of arterioles and venules in the deeper neuropil had no detectable CoA in their vicinity. In the rare instances where CoA were observed in association with deep blood vessels, they did not appear to be preferentially located around arterioles or venules (Fig. 4C,D). Although arterioles and venules located close to the pial surface were more frequently surrounded by CoA (Fig. 4E,F), the correspondence did not appear to be strong, and larger blood vessels such as these were infrequent. Due to the limited number of vessels that had CoA clustered around them, it was not possible to conduct a quantitative analysis of this relationship.

### CoA diameter in relation to natural ageing

The association of patient age with ranked CoA sequence were investigated. There was no significant correlation between age and ranked CoA sequence (*r* = 0.283, *p* = 0.129). Moreover, there was no significant correlation between patient age and CoA density (CoA count or area fraction) across the seven pial surface regions (Table 3). However, the sizes of CoA were found to increase with natural ageing. Patient age was significantly correlated with larger CoA mean and maximum diameters in the fimbria pial surface, prosubiculum pial surface, subiculum pial surface and medial entorhinal cortex pial surface, after FDR correction.

**Table 3:** Spearman's Rho correlation of patient age with tested CoA parameters.

	APS	FPS	PPS	SPS	MPS	LPS	CPS
**CoA count**							
*r*	0.146	− 0.191	− 0.064	− 0.101	− 0.144	0.013	− 0.148
*p*	1	1	0.859	0.892	0.878	0.953	1
*n*	30	30	30	24	24	24	24
**Area fraction**							
*r*	0.188	0.074	0.078	0.107	0.141	− 0.016	− 0.037
*p*	1	0.976	1	1	1	0.940	1
*n*	30	30	30	24	24	24	24
**Mean diameter**							
*r*	0.098	**0.634**	**0.460**	**0.748**	**0.603**	0.020	0.409
*p*	0.776	**0.001**	**0.021**	** < 0.001**	**0.005**	0.946	0.074
*n*	22	**29**	**29**	**23**	**23**	14	23
**Minimum diameter**							
*r*	0.187	0.117	0.081	0.301	0.271	− 0.310	0.299
*p*	0.567	0.637	0.677	1	0.491	0.492	0.578
*n*	22	29	29	23	23	14	23
**Maximum diameter**							
*r*	0.401	**0.596**	**0.437**	**0.679**	0.305	0.530	0.355
*p*	0.090	**0.002**	**0.041**	**0.003**	0.157	0.090	0.113
*n*	22	**29**	**29**	**23**	23	14	23

Significant differences are indicated in bold.

The listed *p* values are after adjustment for Type 1 error with FDR.

*APS* alveus pial surface, *FPS* fimbria pial surface, *PPS* prosubiculum pial surface, *SPS* subiculum pial surface, *MPS* medial entorhinal cortex pial surface, *LPS* lateral entorhinal cortex pial surface, *CPS* collateral sulcus pial surface.

### CoA diameter in relation to obesity

In order to investigate the association of CoA burden with obesity, Spearman's rho correlations were performed between CoA parameters and patients’ BMI, which varied from 20.0 to 42.0 kg/m^2^. No significant correlations were found between ranked CoA sequence and BMI (*r* = 0.201, *p* = 0.325). Moreover, there was no significant correlation between patients’ BMI and CoA density (CoA count and area fraction) or CoA size (CoA mean and minimum diameters) after FDR correction, as shown in Table 4. However, the maximum diameters of CoA were positively correlated with larger BMI in the alveus pial surface, fimbria pial surface and collateral sulcus pial surface.

**Table 4:** Spearman's Rho correlation of patients’ BMI with tested CoA parameters.

	APS	FPS	PPS	SPS	MPS	LPS	CPS
**CoA count**							
*r*	0.315	0.061	0.355	0.515	0.358	0.384	0.316
*p*	0.204	0.769	0.264	0.140	0.170	0.221	0.203
*n*	26	26	26	20	20	20	20
**Area fraction**							
*r*	0.388	0.205	0.410	0.526	0.377	0.346	0.517
*p*	0.088	0.315	0.088	0.121	0.143	0.158	0.069
*n*	26	26	26	20	20	20	20
**Mean diameter**							
*r*	0.193	0.266	− 0.098	0.042	− 0.086	− 0.615	0.436
*p*	0.749	0.465	0.899	0.864	0.847	0.309	0.217
*n*	19	25	25	19	19	11	19
**Minimum diameter**							
*r*	− 0.251	− 0.195	− 0.456	− 0.114	0.002	− 0.367	0.156
*p*	0.698	0.612	0.154	0.748	0.994	0.934	0.732
*n*	19	25	25	19	19	11	19
**Maximum diameter**							
*r*	**0.566**	**0.563**	− 0.123	0.155	0.047	0.404	**0**.**663**
*p*	**0.027**	**0.012**	0.650	0.738	0.847	0.382	**0.014**
*n*	**19**	**25**	25	19	19	11	**19**

Significant differences are indicated in bold.

The listed *p* values are after adjustment for Type 1 error with FDR.

*APS* alveus pial surface, *FPS* fimbria pial surface, *PPS* prosubiculum pial surface, *SPS* subiculum pial surface, *MPS* medial entorhinal cortex pial surface, *LPS* lateral entorhinal cortex pial surface, *CPS* collateral sulcus pial surface.

### CoA diameter in relation to sex

All CoA parameters were compared between male and female groups, but no significant differences were found (as shown in supplementary Fig. S3).

## Discussion

The present study investigated the distribution of CoA in the hippocampus of OSA patients, and found that CoA are concentrated in periventricular regions within an avascular zone that extends 40–100 µm below the pial surface. A spatiotemporal sequence was proposed to describe the progressive spread of CoA from the fimbria through the subpial zone towards more peripheral regions, eventually reaching the collateral sulcus. Importantly patient age and BMI do not correlate with this ranked CoA sequence, nor do the numbers of CoA in the subregions investigated; suggesting that factors other than age or obesity are more influential mediators of CoA deposition. On the other hand, patient age and BMI correlated strongly with the maximum diameters of CoA, supporting the view that CoA increase in size with age and obesity. The implications of these interesting findings are discussed in the following sections.

CoA were detected in 97% (29 out of the 30) of the hippocampal specimens examined in the present study, which is a higher prevalence than reported by other studies: 63% (26 out of 41) of hippocampal sclerosis specimens^29^, 57% (33 out of 58) of patients with mesial temporal lobe epilepsy^17^ and in 52% (24 out of 46) of patients with mesial temporal lobe epilepsy or hippocampal sclerosis^30^. The higher incidence of CoA observed in the present study may be due to methodological considerations, such as the greater sensitivity of fluorescence for detecting CoA, our use of thicker brain sections, and our investigation of more extensive areas of tissue. Alternatively, the higher incidence of CoA in the present study may indicate that OSA favours the deposition of CoA.

The present study found that CoA generally occur in proximity to the lateral ventricle and subarachnoid space. CoA were common in the walls of the lateral ventricle, fimbria and the angle of the CA4 that is closest to the lateral ventricle. This pattern, together with the CoA distribution in seven subpial regions, is consistent with reports that CoA in AD are concentrated in periventricular and subpial regions of the hippocampus^6^. The present study also detected CoA in the pial surface of the medial and lateral entorhinal cortex, which is consistent with a report of CoA in the entorhinal cortex of two AD patients^7^.

In the present study CoA were detected in the CA1 and CA3 in only 10–13% of subjects, and none were observed in the CA2 or subiculum. Thus, apart from the CA4 and fimbria, most of the hippocampus lacked CoA. This distribution differs substantially from that reported by other studies. For example, in three aged healthy subjects and three aged Parkinson’s disease sufferers, CoA were observed in the CA2, as well as in the CA4 of the hippocampus^15^. In another study, CoA were found in the fimbria, alveus, CA3, CA2, CA1 and subiculum of a 39-year-old woman with complex partial seizures^9^. CoA were also observed in the CA4, CA3, CA2, CA1, subiculum and parahippocampal white matter of patients with complex partial seizures and Ammon's horn sclerosis^16^. In contrast, a case report involving a 49-year-old woman with a long history of headaches, observed CoA in the mediotemporal lobe and left parahippocampal white matter but not in cortical gray matter, CA subfields or the dentate gyrus^11^. In view of this variability, it is likely that the distribution of CoA is linked to specific neurodegenerative conditions.

The mean diameter of CoA in the present study was 9.1 ± 0.1 µm and ranged from 6.1 to 15.3 µm; while the CoA minimum and maximum diameters varied from 3.3 to 11.5 µm and 7.7–25.3 µm respectively. These results are consistent with previous reports that the mean diameters of CoA in the human central nervous system range between 4–12 µm and rarely exceed 20 µm^12–14^. In a confocal microscopy ultrastructural study, immature intracellular CoA were reported to be smaller (1.9–11.4 μm), while mature extracellular CoA measured from 4.5 to 18.4 μm^3^.

Although it is generally believed that the numbers of CoA increase with age, with a major increase after 40 years of age^1,12^, the present study found that advancing age is not correlated with CoA density (count or area fraction). Since the majority of the brains investigated in the present study were older than 50 years of age (range 42–89) our results suggest that if a relationship between CoA burden and patient age exists before 50, the effect is lost at later ages. Indeed, literature reports of a positive association between CoA count and patient age are limited to a single study of the retina and optic nerve, where the patients' ages ranged from 26 to 83 years^13^, while a study of an older cohort aged 46–84 years was unable to find a significant correlation^14^.

An unexpected finding to emerge from the present study was that the distribution of CoA in subpial regions of the hippocampus conformed to a pattern: when few CoA were present, they were invariably concentrated in and around the fimbria, and as the number of CoA increased, they systematically extended further away from the fimbria and were smaller in size. Thus, the packing density and size of CoA peaked in the fimbria while adjacent regions displayed a progressive reduction in the density and mean diameter of CoA, specifically, the alveus on one side and the prosubiculum, subiculum, medial entorhinal cortex, lateral entorhinal cortex and collateral sulcus on the other side. Moreover, the CoA maximum diameter in the fimbria was consistently greater than in adjacent regions, and the smallest mean and maximum CoA diameters were found in the collateral sulcus. Since CoA size was positively correlated with patient age, the presence of larger CoA in the fimbria supports the idea that the fimbria is the first site of CoA accumulation. The smaller CoA in the collateral sulcus are consistent with a delay in CoA deposition, as the CoA gradually spread from the fimbria along the pial border to the collateral sulcus. If the most recently generated CoA have the smallest diameters, then the presence of the smallest minimum diameters in the fimbria (Table 2) suggest that this region is the most active site of CoA generation. We speculate that the positive correlation between patient age and CoA diameter indicates that once formed, CoA remain for a long time, perhaps for several decades. During this time they may gradually grow in size by fusion or by absorbing additional materials.

One of the dominant hypotheses concerning the nature of CoA is that they are waste containers that are trafficked from the brain into the blood^5^. Three lines of evidence from the present study do not support this idea. First, CoA were not commonly observed in proximity to the perivascular space around blood vessels. Only a small number of hippocampal specimens in the present study exhibited CoA near the walls of any blood vessels. These sporadic vessels had large diameters and were located in the deep white matter. Second, in the rare instances where CoA were observed in the perivascular space, they were observed equally often around arterioles and venules. Since only venules, with their thinner walls and lower blood pressure convey waste from the brain, the lack of a preferential distribution around venules argues against the vascular waste disposal hypothesis. Third, CoA are most prevalent in the subpial zone, within 100 µm or so beneath the pial surface. It is notable that immunocytochemical labelling of adjacent brain sections revealed a paucity of microvessels in this subpial region (Fig. 4A,B). Taken together, these three sets of observations make it unlikely that CoA are cleared into the bloodstream, unless the clearance rate is highly efficient and results in the rapid removal of CoA from the perivascular space.

Our finding that most CoA are located within 100 µm of the lateral ventricle or subarachnoid space (adjacent to the pia) is intriguing, as in living brains both of these spaces are filled with CSF. This correspondence raises the possibility that CoA may interact with the CSF, or as several authors have speculated, CoA may originate from exudates of the CSF^10,31–34^. A recent study reported the presence of CoA in the CSF and cervical lymph nodes, and proposed that CoA may serve as containers that remove waste from the brain via the CSF^35^. Several lines of evidence from the present study support this novel idea. CoA were more numerous and larger near the pial surface (Fig. 3), and individual CoA were sometimes observed on the pial surface (Fig. 1B), within the ventricular cavity (Fig. 1C). Furthermore, it is known that the rates of CSF production and absorption slow down significantly with natural ageing^36–39^. Such a reduction in CSF flow might slow the clearance of CoA and cause them to remain for longer in the adjacent neuropil, leading to an age-related increase in the average size of CoA.

Due to the retrospective nature of the autopsy series used, the present study has several limitations, specifically: (i) a moderate sample size: 30 OSA patients; (ii) the absence of a healthy age-matched control group: the present study was part of a larger project that examined neuropathological changes in OSA patients, and we had no access to tissue from healthy controls; (iii) the limited number of sections available prevented stereological analysis, which is most accurate way to quantify diameters. Nevertheless, as all tissues were analysed in exactly the same way, the differences observed in CoA diameters between regions or brains are valid, although they are relative differences rather than absolute ones. These limitations do not weaken the spatio-temporal progression of CoA reported here.

In conclusion, the present study has extended our understanding of CoA in the human hippocampus. Our data indicate that while CoA become larger with age, the hippocampal CoA burden does not increase beyond 50 years of age. Our data are not consistent with the prevailing notion that CoA are disposed of via the vasculature. Instead, we propose that hippocampal CoA originate from the vicinity of the fimbria and then spread through the subpial zone into adjacent regions, until they are released into the CSF.

## Supplementary Information

Supplementary Information.
