# Supplementary material for: Quantitative analysis of size and regional distribution of corpora amylacea in the hippocampal formation of obstructive sleep apnoea patients
Source: Sci Rep. 2021 Oct 22;11:20892. doi: 10.1038/s41598-021-99795-8 (PMC8536671; doi:10.1038/s41598-021-99795-8)
Supplement: Supplementary file 1 — Supplementary Information. [file 41598_2021_99795_MOESM1_ESM.pdf]

Title Page (Supplementary information)

Quantitative analysis of size and regional distribution of corpora amylacea in the hippocampal formation of obstructive sleep apnoea patients

Cuicui Xu[1], Jessica E. Owen[1], Thorarinn Gislason[2,3], Bryndis Benediktsdottir[2,3], Stephen R. Robinson[1,4]*

[1] School of Health and Biomedical Sciences, RMIT University, Bundoora, Victoria, Australia.

[2] Department of Respiratory Medicine and Sleep, Landspitali – The National University Hospital of Iceland, Reykjavik, Iceland.

[3] Department of Clinical Biochemistry, Landspitali – The National University Hospital of Iceland, Reykjavik, Iceland.

[4] Institute for Breathing and Sleep, Austin Health, Heidelberg, Victoria, Australia.

***Corresponding author:**

Professor Stephen R. Robinson

School of Health and Biomedical Sciences

College of Science, Engineering & Health

RMIT University, PO Box 71

Bundoora, VIC. 3083, Australia

Tel: +613 9925-7120

Fax: +614 0457-8383

Email: stephen.robinson@rmit.edu.au

Supplementary Table 1 Immunohistochemistry dilution protocol.

	Blocking (3 h)	Primary (18 h)	Secondary (3 h)	Tertiary (3 h)
1% BSA	✓	✓	✓	✓
4% serum[1]	✓	✓	✓	
1% Triton X-100	✓	✓		
1% Ethanolamine	✓			
1st antibody		✓[2]		
2nd antibody			✓	
SB-HRP				✓

[1]Serum from the host animal of 2nd antibody was used in the dilution. [2]Control slides were incubated without primary antibody.

Supplementary Table 2 Hippocampal subregions examined in this study, showing the proportion of brain samples that contained CoA.

**Region**	**LV**	**Fimbria**	**CA4**	**CA3**	**CA2**	**CA1**	**Subiculum**
Number (* / #)	23 / 24	29 / 30	19 / 30	3 / 30	0 / 30	4 / 30	0 / 30
Percentage (%)	95.8	96.7	63.3	10.0	0	13.3	0
**Region**	**APS**	**FPS**	**PPS**	**SPS**	**MPS**	**LPS**	**CPS**
Number (* / #)	22 / 30	29 / 30	29 / 30	23 / 24	23 / 24	14 / 24	23 / 24
Percentage (%)	73.3	96.7	96.7	95.8	95.8	58.3	95.8

* indicates the number of brains that contained CoA; # indicates the number of brains examined; % indicates the proportion of examined brains that contained CoA. LV: wall of lateral ventricle; APS: alveus pial surface; FPS: fimbria pial surface; PPS: prosubiculum pial surface; SPS: subiculum pial surface; MPS: medial entorhinal cortex pial surface; LPS: lateral entorhinal cortex pial surface; CPS: collateral sulcus pial surface.

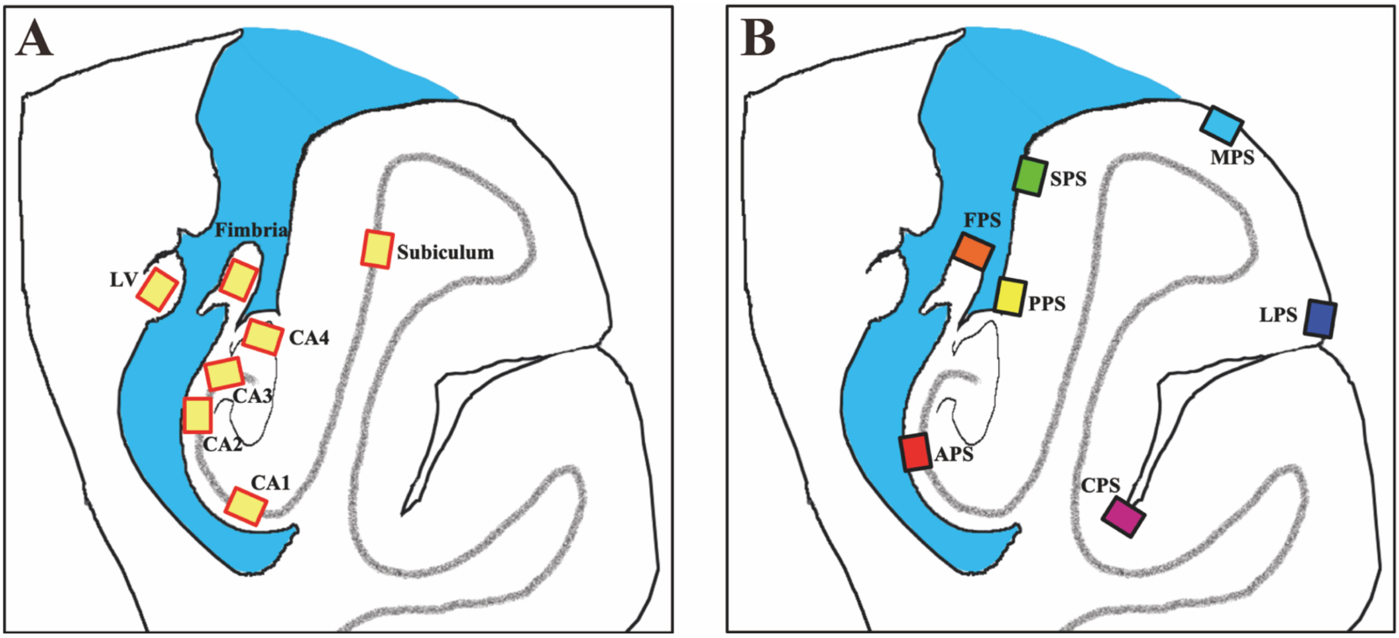

Supplementary Figure 1. A, Seven hippocampal neuropil regions that surround lateral ventricle (the blue area) include: LV (wall of lateral ventricle), fimbria, CA4, CA3, CA2, CA1 and subiculum. B, Seven pial surface regions investigated: APS (alveus pial surface), FPS (fimbria pial surface), PPS (prosubiculum pial surface), SPS (subiculum pial surface), MPS (medial entorhinal cortex pial surface), LPS (lateral entorhinal cortex pial surface) and CPS (collateral sulcus pial surface). The coloured rectangles indicate where micrographs were taken for the analysis of CoA packing density. These regions were chosen because they are spaced across the hippocampal formation, and they can be reliably identified in sections due to their proximity to landmarks.

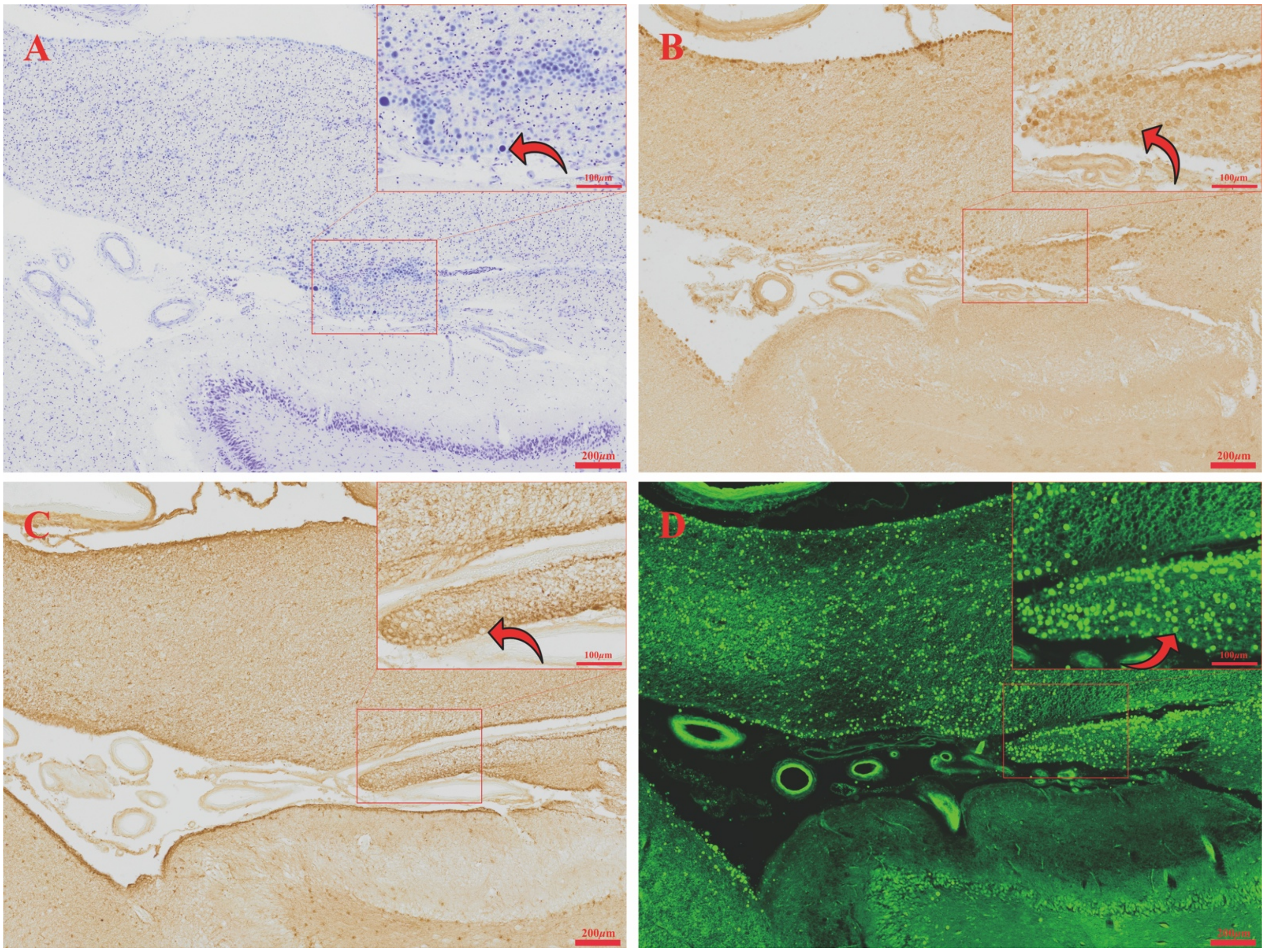

Supplementary Figure 2. CoA appearance from the same subject were revealed by four histological staining methods. A, Cresyl violet (0.5%). B, Immunohistochemistry with anti-Tau (1:500); C, anti-GFAP (1:20,000); D, Control (without primary antibody) under fluorescent mode (FITC). CoA can be seen easily with cresyl violet (A) and tau (B) as the objects stand out against a faint background. Comparatively the image stained with GFAP (C) show the coats of a small subpopulation of CoA but do not label their contents, leaving a 'hole' in the background labelling to signify the location of the CoA. Individual CoA are indicated by arrows. It is possible that the CoA in (B) and (C) are indicated by immunoglobulins rather than by antibodies to GFAP and tau. CoA were best detected under fluorescent illumination with control slides of immunohistochemistry (D) the signal was easy to distinguish from background and there was no other significant staining to obscure the detecting of CoA. Hence all of the analyses of CoA in this study are based on the fluorescent images.

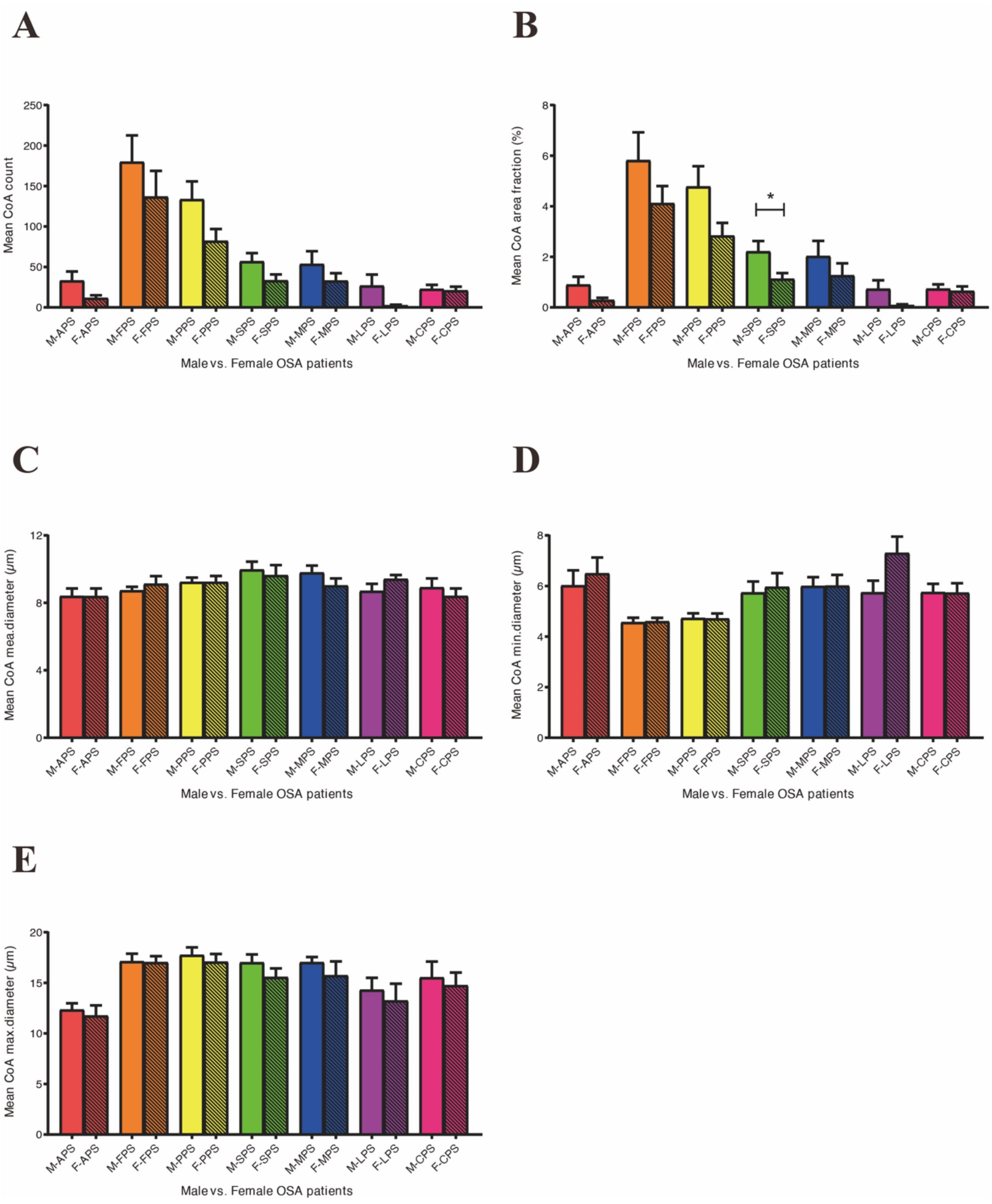

Supplementary Figure 3. The association of CoA progression with sex. CoA parameters in the male (blank bars) and female OSA (striped bars) groups in seven pial surface regions of CoA count (A), CoA area fraction (B), CoA mean diameter (C), CoA minimum diameter (D) and CoA maximum diameter (E). Unpaired 2-tailed *t*-tests between male *vs.* female OSA groups. Mean ± SEM. APS: alveus pial surface, FPS: fimbria pial surface, PPS: prosubiculum pial surface, SPS: subiculum pial surface, MPS: medial entorhinal cortex pial surface, LPS: lateral entorhinal cortex pial surface and CPS: collateral sulcus pial surface.

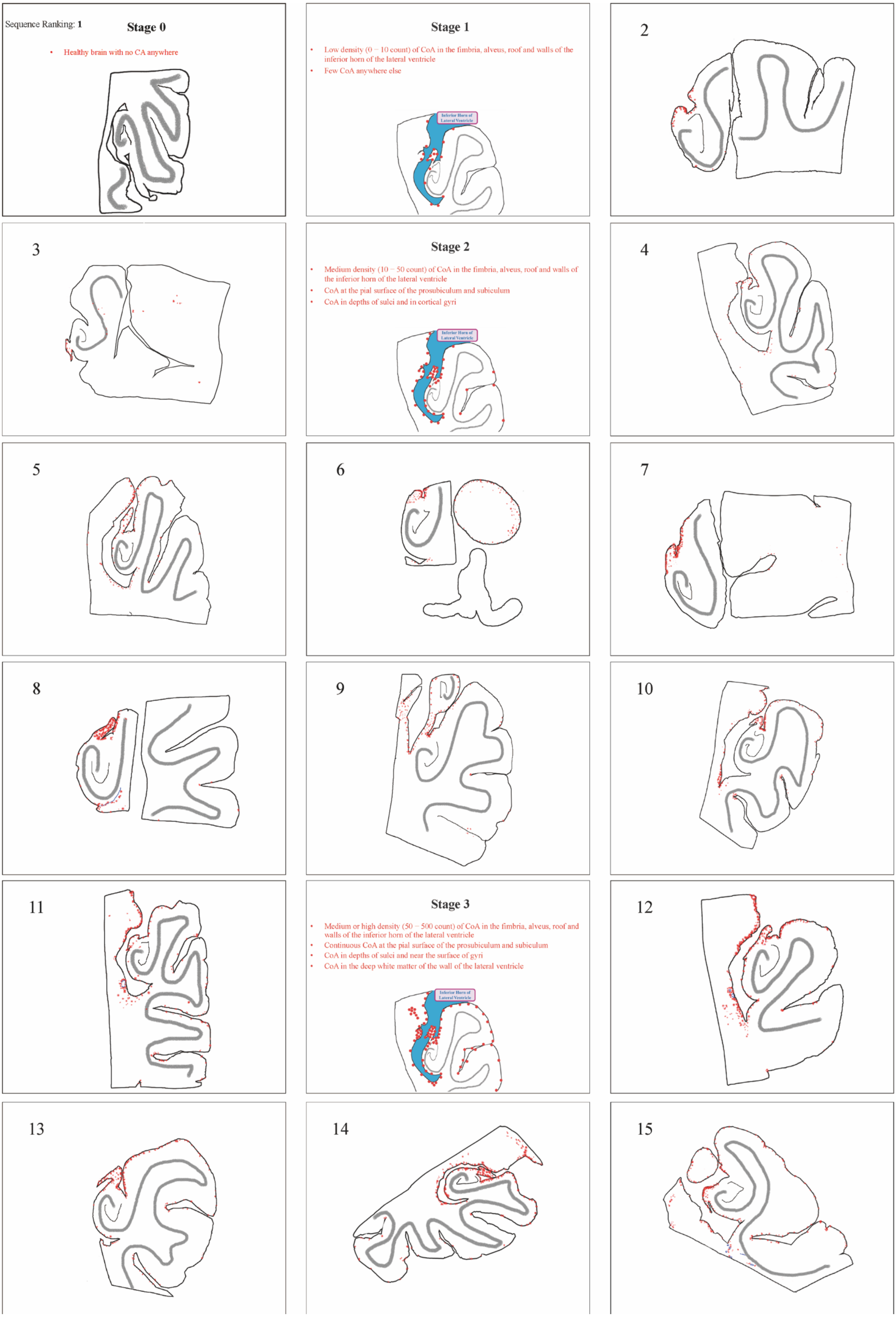

Sequence Ranking: 1
Stage 0
Healthy brain with no CA anywhere
Stage 1
Low density (0 – 10 count) of CoA in the fimbria, alveus, roof and walls of the inferior horn of the lateral ventricle
Few CoA anywhere else
Inferior Horn of Lateral Ventricle
2
3
Stage 2
Medium density (10 – 50 count) of CoA in the fimbria, alveus, roof and walls of the inferior horn of the lateral ventricle
CoA at the pial surface of the prosubiculum and subiculum
CoA in depths of sulci and in cortical gyri
Inferior Horn of Lateral Ventricle
4
5
6
7
8
9
10
11
Stage 3
Medium or high density (50 – 500 count) of CoA in the fimbria, alveus, roof and walls of the inferior horn of the lateral ventricle
Continuous CoA at the pial surface of the prosubiculum and subiculum
CoA in depths of sulci and near the surface of gyri
CoA in the deep white matter of the wall of the lateral ventricle
Inferior Horn of Lateral Ventricle
12
13
14
15

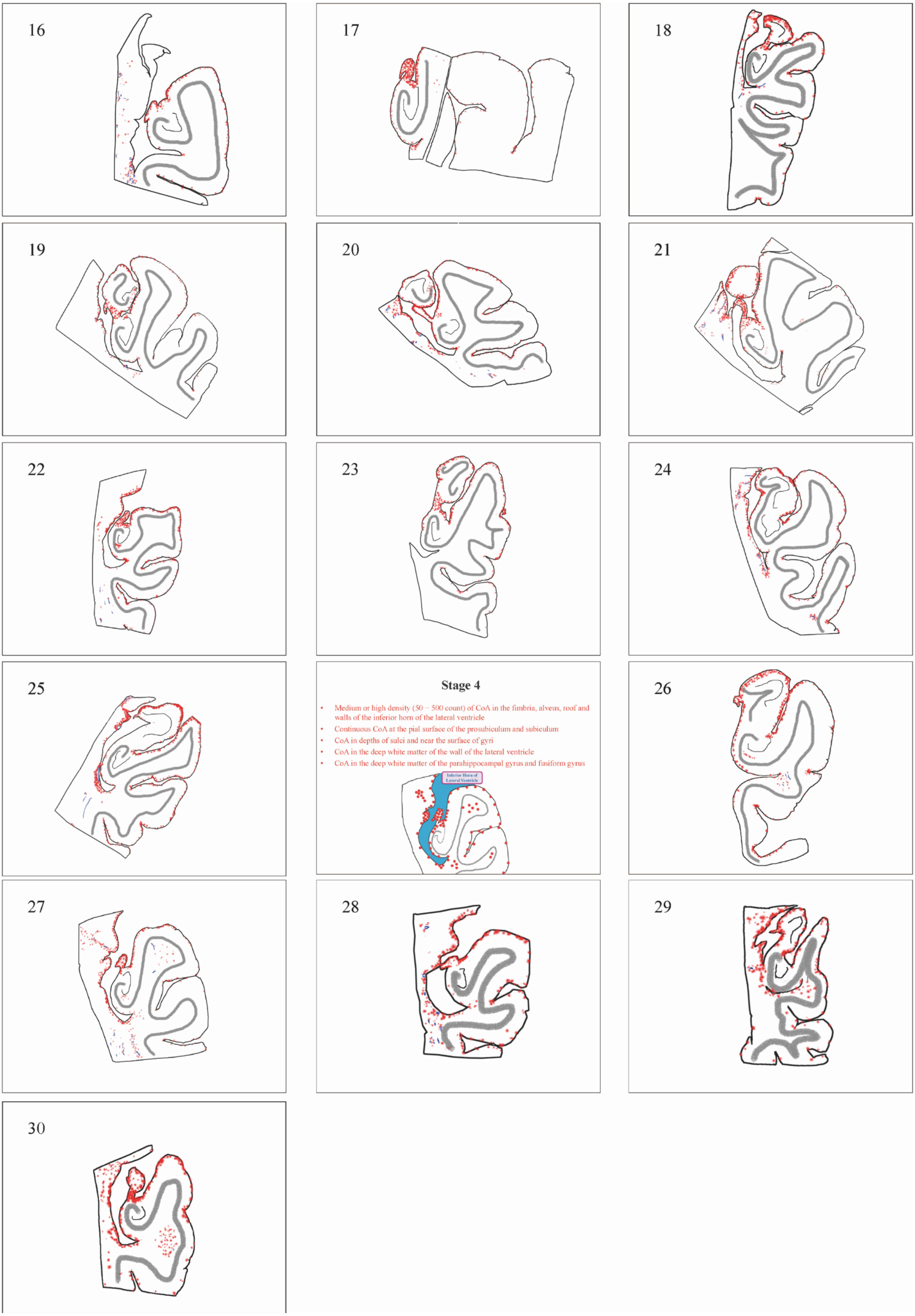
16
17
18
19
20
21
22
23
24
25
Stage 4
• Medium or high density (50 – 500 count) of CoA in the fimbria, alveus, roof and walls of the inferior horn of the lateral ventricle
• Continuous CoA at the pial surface of the prosubiculum and subiculum
• CoA in depths of sulci and near the surface of gyri
• CoA in the deep white matter of the wall of the lateral ventricle
• CoA in the deep white matter of the parahippocampal gyrus and fusiform gyrus
Inferior Horn of Lateral Ventricle
26
27
28
29
30
